# Autism Cognizance: A dilemma among medical and Allied Medical practitioners

**DOI:** 10.12669/pjms.36.4.1703

**Published:** 2020

**Authors:** Naheed Akhter, Nazia Mumtaz, Ghulam Saqulain

**Affiliations:** 1 Ms. Naheed Akhter, M. Phil (Speech Language Pathology), Head of Occupational Therapy, Autism Resource Centre, Rawalpindi, Pakistan; 2 Dr. Nazia Mumtaz, PhD (Rehabilitation Sciences), Head of Department, Isra Institute of Rehabilitation Sciences, Isra University, Islamabad, Pakistan; 3 Dr. Ghulam Saqulain, F.C.P.S (Otorhinolaryngology), Head of Department Otorhinolaryngology, Capital Hospital, Islamabad, Pakistan

**Keywords:** Autism spectrum disorder, Awareness, Knowledge, KCAHW, Occupational therapist, Pediatricians, Physicians

## Abstract

**Objectives:**

To determine the awareness about autism among Medical and Allied-Medical Practitioners.

**Methods:**

This cross-sectional survey recruited a sample of n=300 participants including n=150 Medical and n=150 Allied-Medical practitioners using convenience sampling from Benazir Bhutto Hospital and Autism Resource Centre, Rawalpindi, Pakistan between 20^th^ May 2018 to 20^th^ October 2018. Sample included both genders, aged 21 to 50 years. Basic demographic sheet and Knowledge about Childhood Autism among Health Workers (KCAHW) Questionnaire was used to collect data. Statistical analysis was done using SPSS 21.

**Results:**

Study revealed the total mean KCAHW score of 15.20 ± 5.17 and 8.84 ± 6.31 for Allied-Medical and Medical practitioners respectively. While the Domain-I mean KCAHW scores was 6.28±2.10 and 3.68±2.41; Domain-II score 0.86 ± 0.35 and 0.45 ± 0.50; Domain-III score 3.28 ± 1.00 and 1.91 ± 1.65; and Domain-IV score of 4.83 ±1.72 and 2.80 ± 1.75 for Allied-Medical and Medical practitioners respectively.

**Conclusion:**

The present study shows that there is significant awareness regarding autism among Allied-Medical compared to Medical practitioners.

## INTRODUCTION

Autism spectrum disorders (ASD) is an umbrella for a number of conditions including Autism.^1^ Starting in the first three years of life it is diagnosed with the presence of two criteria including disorders in social interaction and communication; and abnormal repetitive behavior, interests and activities.^1^ ASD is a common pathology with a prevalence among 8-year-old children alone being 13.1 to 29.3 per 1000, with a male female ratio of 4:1.^2^ According to Mason D et al. ASD sufferers have lower Quality of life.^3^ Deficiency of knowledge about Autism among doctors and failure to guide caregivers may result in delay in both diagnosis and subsequent early evidence-based intervention well-known to be beneficial.^4^ Early specialized multidisciplinary care to facilitate positive results and improved educational and behavioral outcomes, characterize the treatment of autism. The gap in the awareness and knowledge of among health care providers can result in diagnostic and interventional delays with an interesting fact being that young children most frequently visit medical practitioners and not allied practitioners. Lack of access to various health care options, financial burden with lack of insurance cover, increase family impact and burden.^5^

Deficient evidence regarding awareness about ASD among medical and allied professionals in local literature exist. Although Pediatricians play an important role to guide families with the average age of diagnosis of ASD of 52 months in United States.^2^

Elsabbagh M et al. has suggested a critical requirement for conducting research and advocated capacity building for low and middle income countries to cater to Autism.^6^ In the context of being a developing country with a population of around 220 million with 45% population below 18, we expect a worse scenario. The true current local prevalence of ASD in children is unknown, however a prevalence of 8.6 has been reported among adult patients who reported to a hospital.^7^,^8^ Another local study among healthcare professionals about Autism revealed misconceptions and unbalanced understanding^9^, and similar situation among general practitioners.^10^ Therefore this study was conducted with the objective to determine the awareness about autism among Medical and Allied-Medical practitioners.

## METHODS

This Cross-Sectional study recruited a sample of n=300 participants divided into two groups including 150 Medical and 150 Allied-Medical practitioners using convenience sampling. These included both genders aged 21-50 years from Benazir Bhutto Hospital and Autism Resource Centre, Rawalpindi, Pakistan from 20th May 2018 to 20th October 2018. Practitioners with just one-year experience and Homoeopaths were excluded from the study. The study was conducted after ethical approval (Ref. No. 1502-M.Phil SLP-002, dated April 18th, 2017) of research from Advanced Studies and Research Committee, ISRA University, Islamabad, Pakistan.

Basic demographic sheet and Knowledge about Childhood Autism among Health Workers (KCAHW),^11^ were used for data collection. KCAHW is a 19 item self-administered questionnaire divided into four Domains. Each questions have three options with the correct option scored as one. Domain-I contains eight questions addressing impairments in social interaction. Domain-II contains only one question addressing impairment in area of communication and language development. Domain-III contains four questions addressing area of obsession and compulsive pattern of behavior. Domain-IV contains six questions addressing information on type of disorder is childhood autism, possible co-morbidities and onset of childhood autism. The mean total score is a measure of level of knowledge about childhood autism. Following informed consent, Basic demographic sheet and KCAHW questionnaire was applied to the study population by the researcher. SPSS 21.0 was used for data analysis. For Qualitative data like gender frequency and percentage were calculated. Variables specially studied included mean and standard deviation of KCAHW Total and Domain Score. T-test was used to see any statistically significant difference between the groups, followed by comparison of results with local and international literature.

## RESULTS

The results of the current study revealed that the study population of two groups Medical and Allied Medical practitioners (n=150 each) comprised of a male population of 100 (33.3%) and female population of 200 (66.7%), and age ranging from 21-50 years. Among the Medical practitioners, majority 53.34% were general practitioners while in the Allied-Medical group majority 46.67% were psychologists. As regards service experience, majority 37.35 had 6-10 years’ experience (Table-I).

**Table-I:** Frequency distribution & mean KCAHW Questionnaire score for different experience and specialities Of Allied Medical & Medical And Anova Statistics (n=300).

Experience (n=300)				Allied Medical (n=150)			Medical (n=150)		

Years	N, %		Mean	Specialties	N, %	Mean	Specialties	N	Mean*
1-5	33, 11%		0.5139	Occupational Therapist	10 6.66%	0.6380	Medical specialist	8, 5.33%	0.2891
6-10	112, 37.35%		0.6104	SLP	40 26.67%	0.7442	Medical Officer	5, 3.33%	0.3060
11-15	78, 26%		0.6846	Physiotherapist	30, 20%	0.7965	Psychiatrist	36, 24%	0.4558
16-20	65, 21.7%		0.6308	Psychologist	70, 46.67%	0.8562	General practitioner	80, 53.34%	0.4644
21-25	12, 4%		0.6181				Pediatrician	21, 14%	0.4649
ANOVA	F= 1.803 P= 0.128			F= 4.796 P= 0.003				F= 0.902 P= 0.465	

***Note:*** Means for groups in homogeneous subsets are displayed.

In the present study the total KCAHW mean score obtained was 15.20 ± 5.17 and 8.84 ± 6.31 for Allied-Medical and Medical practitioners respectively (Table-II). The Domain wise mean KCAHW scores included for the Domain-I, 6.28±2.10 and 3.68±2.41; for Domain-II, 0.86 ± 0.35 and 0.45 ± 0.50; for Domain-III, 3.28 ± 1.00 and 1.91 ± 1.65; and for Domain-IV, 4.83 ±1.72 and 2.80 ± 1.75 for Allied-Medical and Medical practitioners respectively (Table-II).

**Table-II:** Comparison of mean & total domain scores on KCAHW questionnaire among Allied-Medical and Medical Practitioners & Independent Sample t-test Statistics (n=300).

Domain	Qualification	Mean	Std. Deviation	t-test for Equality of Means	

				t	P-value
Domain1 (8)	Allied-Medical	6.23	2.10	10.184	0.000
	Medical	3.68	2.41	10.184	0.000
Domain-II (1)	Allied-Medical	0.86	0.35	8.135	0.000
	Medical	0.45	0.50	8.135	0.000
Domain-III (4)	Allied-Medical	3.28	1.00	8.785	0.000
	Medical	1.91	1.65	8.785	0.000
Domain-IV (6)	Allied-Medical	4.83	1.72	7.919	0.000
	Medical	2.80	1.75	7.919	0.000
Total	Allied-Medical	15.20	5.17	11.455	0.000
	Medical	8.84	6.31	11.455	0.000

There was significant relationship between the total mean score on the KCAHW questionnaire (t=11.45, df= 298, p=.000) and Allied-Medical practitioners scored 15.20 ± 5.17 as against means score 8.84 ± 6.31 by the Medical practitioners. The Allied-Medical practitioners also scored higher scored then the Medical practitioners in all the four domains.

Experience distribution revealed highest mean score for participants with experience of 11 to 15 years while least score was observed in 1-5 years’ experience group. However one way Anova with P-value of 0.128 did not indicate statistically significant difference. In Allied Medical group psychologists showed the maximum score followed by physiotherapist and the difference was statistically significant with p-value of 0.003. In the Medical group highest mean score was noted in Pediatricians followed by General practitioners and lowest in Medical Specialists with p-value of 0.465 (Table-I).

## DISCUSSION

In the present study the total KCAHW mean score obtained by Allied–Medical practitioners was 15.20 ± 5.17 as against 8.84 ± 6.31 for Medical practitioners which was statistically significant indicating significantly better knowledge of Autism in Allied-Medical practitioners compared to Medical Practitioners. Similarly, in a local study by Rahbar MH et al. reported poor knowledge of autism in General Practitioners with only 44.6% reportedly heard of autism.^10^ In another study in Lahore, Non-Physicians were reportedly more likely to identify Autism with p<0.001.^9^ In contrast to our study Eseigbe EE et al. in a Nigerian study involving medical doctors, revealed that Pediatricians, psychiatrist and those working in tertiary hospital had good knowledge with KCAHW Score > 15, which was statistically significant,^12^ A Turkish study revealed that in the first grade nursing and medical students 8% were highly aware, 70.9% moderately and 21.1% not aware about autism.^13^

In the current study, the Allied-Medical practitioners also scored higher than the Medical practitioners in all the four domains of KCAHW with mean scores for the Domain-I being 6.28±2.10 and 3.68±2.41; for Domain-II), 0.86 ± 0.35 and 0.45 ± 0.50; for Domain-III, 3.28 ± 1.00 and 1.91 ± 1.65; and for Domain-IV, 4.83±1.72 and 2.80± 1.75 for Allied-Medical and Medical practitioners respectively.

In a SirLankan study, involving doctors reported that 61.9% had low knowledge of autism. However, knowledge was high for Domain-I and lowest for Domain-III.^14^ In yet another study Eseigbe EE et al. medical doctors including Pediatricians, Psychiatrist and doctors working in tertiary hospital showed good knowledge of Autism with best scores reported for Domain-II and least in Domain-IV.^12^ In contrast in the present study highest score achieved by Medical practitioners was in Domain-III and lowest in Domain-II. While in an Egyptian study Hend MS reported dearth of knowledge among family physicians with a total mean KCAHW score of 11.2 ±3.5, with highest score in Domain-I.^15^

Allied-Medical Practitioners achieved a high KCAHW total mean score of 15.20±5.17 in the current study and the highest score were achieved by them in Domain-II and lowest in Domain-I. In a Nigerian study involving teachers a low knowledge of ASD with a total mean score of 10.81 ± 4.13 with a slightly higher score for urban teachers (11.21 ± 4.31) was reported.^16^ In contrast study by Sampson and Sandra^17^, psychiatry nurses had more knowledge of ASD compared to pediatric nurses, however both had low knowledge.

In the current study, the Allied-Medical practitioners achieved higher score than the Medical practitioners in all the four domains. In contrast in an Iranian study by Effatpanah M et al. involving pediatricians and health care workers, 98.6% reported knowledge of autism and no significant difference was noted in rating DSM-IV TR for diagnosis of ASD.^18^

Study also revealed highest mean score for participants with experience of 11 to 15 years, while least score was observed in 1-5 years’ experience group. However difference was not statistically significant. Similarly, in a study by Hend MS, targeting family physicians, those with previous experience had higher scores.^15^ In contrast in a local study to access the knowledge of general practitioners, it was reported that GP’s who had acquired their medical degrees only 1-5 years back reported better knowledge of autism compared to older’s^10^, indicating need for continuing medical education in the country.

Present study revealed that in the Allied Medical group, psychologists had the maximum score followed by physiotherapist and the difference was statistically significant. In another study, better knowledge was reported in psychiatry nurses compared to pediatric nurses, though both had low knowledge.^17^ In current study nurses were not the target of study.

In the Medical group, the present study revealed highest mean score was in Pediatricians followed by General practitioners and lowest in Medical Specialists however this was not statistically significant. Similarly, Good knowledge was also noted in pediatricians in a study by Effatpanah M et al.^18^ In a study by Eseigbe EE et al. higher score was noted in pediatricians and psychiatrist compared to GP’s.^12^

An important factors which need to be catered to in development and provision of health care for ASD, include creation and plan interventions, share resources and services information and collaboration in the health care field and research.^19^ Hence it is essential for provision of health care for Autistic children to equip Medical and Allied-Medical practitioners with requisite knowledge, especially keeping in view the dearth of knowledge of autism as pointed out in this study for Medical practitioners.

### Limitations of the study

Due to small sample size and limitation of study to Rawalpindi and Islamabad region, this study lacks generalizability. Moreover, this study did not target the nurses since they are generally not responsible for counselling parents of Autistic children in this region.

## CONCLUSIONS

The present study shows that there is significant awareness regarding autism among Allied-Medical compared to Medical practitioners.

### Author`s Contribution

**NA:** Conceptualization of work, designing of research methodology.

**NM:** Data analysis and interpretation, Critical Revision.

**GS:** Manuscript preparation, literature review and final publication and responsible for the accuracy of study.
